# Intelligent Virtual Sensor Generation Using KL-Divergence- Based Fusion and Deep Generative Learning for Smart Environmental Monitoring

**DOI:** 10.3390/s26134123

**Published:** 2026-06-30

**Authors:** Murad Ali Khan, Qazi Waqas Khan, Muhammad Faizan, Ji-Eun Kim, Il-yeop Ahn, Do-Hyeun Kim

**Affiliations:** 1 Department of Computer Engineering, Jeju National University, Jeju 63243, Republic of Korea; muradali@stu.jejunu.ac.kr (M.A.K.);; 2 Autonomous IoT Research Center, Korea Electronics Technology Institute, Seongnam 13509, Republic of Korea

**Keywords:** smart sensors, virtual sensors, intelligent sensing, sensor data fusion, KL divergence, deep generative learning, variational autoencoder, conditional tabular GAN, machine learning for sensor data analysis, BiLSTM, BiGRU, environmental monitoring

## Abstract

Sensor-based environmental monitoring systems are often affected by missing, noisy, and unreliable measurements caused by sensor faults, sparse deployment, calibration drift, and communication interruptions. To address these challenges, this study proposes an intelligent virtual sensor generation framework that integrates physical-constraint-based preprocessing, statistical virtual sensor modeling, KL-divergence-based fusion, deep generative augmentation, and temporal prediction. The raw weather-station data are first refined using threshold-based filtering, physical validity constraints, and Isolation Forest-based outlier detection. To handle the circular nature of wind direction, the angle is encoded using sine and cosine components during modeling and reconstructed using the atan2 function for evaluation. Multiple statistical methods, including Inverse Distance Weighting, Kernel Density Estimation, Ridge Regression, and Copula-based modeling, are employed to generate complementary virtual sensor data. These outputs are adaptively fused using KL divergence according to their distributional similarity with real sensor data. The fused datasets are further augmented using Variational Autoencoders and Conditional Tabular Generative Adversarial Networks, and then evaluated using BiLSTM and BiGRU models with MAE, MSE, and RMSE metrics. The experimental results demonstrate that the proposed framework generates physically valid and distributionally consistent virtual sensor data. Fusion-based methods outperform standalone approaches, while VAE-based augmentation generally provides better statistical fidelity and lower prediction errors than CTGAN. Additional validation using a public NOAA weather-station dataset further supports the transferability of the proposed fusion-based virtual sensing workflow. Comparisons with TimeGAN and diffusion-based temporal generative baselines, supported by Wilcoxon signed-rank testing, confirm the statistical significance and competitive performance of the proposed framework. A quantitative computational analysis also demonstrates the practical feasibility of the framework in terms of training time, inference time, memory consumption, and scalability. Overall, the proposed framework offers a reliable and scalable solution for virtual sensing in sensor-sparse and fault-prone environmental monitoring systems.

## 1. Introduction

The rapid advancement of sensor-based monitoring systems across environmental, industrial, and energy domains has generated large-scale, high-frequency data streams. Despite these developments, real-world deployments remain susceptible to several practical challenges, including sensor failures, calibration inconsistencies, cost constraints, and data loss due to network or device malfunctions. Such limitations not only compromise data quality but also adversely affect the reliability of downstream data-driven decision-making systems. In this context, virtual sensor machine learning models designed to emulate the behavior of physical sensors have emerged as a promising solution, enabling the estimation of missing or unreliable measurements with high accuracy [1,2]. However, developing high-fidelity virtual sensors requires a systematic pipeline that encompasses rigorous preprocessing, robust data-generation mechanisms, and comprehensive validation strategies to ensure alignment with real-world system dynamics.

A fundamental prerequisite for reliable virtual sensor modeling is effective data preprocessing, as raw sensor data are often contaminated with noise, outliers, and missing values. Traditional approaches such as threshold-based filtering, alongside advanced techniques like Isolation Forest, play a critical role in detecting anomalies, particularly in high-dimensional and multivariate time-series datasets [3]. Additionally, missing value imputation must be handled with caution to prevent the introduction of bias that could propagate through subsequent modeling stages. Without adequate preprocessing, even sophisticated machine learning models may produce inaccurate or misleading predictions, especially in mission-critical applications such as environmental monitoring and smart grid management [4].

To address data incompleteness, synthetic or virtual sensor data can be generated using a variety of statistical and spatial modeling techniques. Methods such as copula-based simulations [5], regression-based modeling [6], and spatial interpolation techniques like Inverse Distance Weighting (IDW) enable the realistic emulation of sensor behavior under diverse operating conditions [7]. In particular, copula models are highly effective in capturing complex dependencies among multiple variables, making them well-suited for multivariate sensor data generation [8]. Nonetheless, ensuring the statistical fidelity of generated data remains a critical challenge. Techniques such as Kernel Density Estimation (KDE) and constraint-based validation, grounded in sensor specifications, are commonly employed to assess the consistency of synthetic data with real-world distributions and physical laws [9].

An essential component of improving virtual sensor data quality is data fusion [10], wherein outputs from multiple generation methods are integrated to leverage their complementary strengths. By utilizing Kullback–Leibler (KL) divergence as a measure of distributional similarity [11], synthetic datasets generated from different approaches can be adaptively weighted and combined based on their closeness to real sensor data. This fusion strategy enhances data realism, mitigates individual model biases, and improves the generalization capability of downstream learning models [12]. Consequently, the fusion process enables the construction of more robust and representative virtual sensor datasets.

To further enrich the dataset and enhance model robustness, data augmentation techniques based on deep generative models are employed. Variational Autoencoders (VAE) [13] and Conditional Tabular Generative Adversarial Networks (CTGAN) [14] are particularly effective for tabular sensor data, as they can learn complex data distributions and generate high-quality synthetic samples. These models have demonstrated significant improvements in generalization performance, especially in data-scarce scenarios [15,16]. The augmented datasets are subsequently utilized to train advanced sequence learning models such as Bidirectional Long Short-Term Memory (BiLSTM) and Bidirectional Gated Recurrent Units (BiGRU), which are capable of capturing intricate temporal dependencies in sensor data [17]. The performance of the proposed framework is evaluated using standard regression metrics, including Mean Squared Error (MSE), Root Mean Squared Error (RMSE), and Mean Absolute Error (MAE), providing quantitative validation of its effectiveness [18,19].

Overall, the proposed framework presents a comprehensive pipeline that integrates preprocessing, statistical data generation, distribution-aware fusion, deep generative augmentation, and temporal modeling to develop reliable and scalable virtual sensors for real-world applications. Unlike existing approaches that mainly focus on isolated prediction, interpolation, or synthetic data generation tasks, this study further validates the proposed virtual sensing framework against temporal generative baselines and statistically verifies the observed performance differences using paired significance testing.

**Key Contributions:**

- Unified Framework: An end-to-end architecture integrating statistical modeling and deep learning for robust virtual sensor development.
- KL-based Data Fusion: A distribution-aware fusion mechanism leveraging KL divergence to combine multiple synthetic data sources.
- Validation Strategy: A hybrid validation approach using KDE analysis and sensor-based physical constraints to ensure data realism.
- Advanced Data Augmentation: Integration of VAE and CTGAN to enhance data diversity and improve model generalization.
- Comprehensive Evaluation: Performance validation using BiLSTM and BiGRU models with standard regression metrics, demonstrating high predictive accuracy.
- Baseline and Statistical Validation: Comparative evaluation against TimeGAN and diffusion-based temporal generative baselines, supported by Wilcoxon signed-rank testing to verify the statistical significance of performance differences.

## 2. Related Work

The widespread adoption of sensor technologies in smart environments, spanning environmental monitoring, industrial automation, and energy systems, has significantly increased interest in virtual sensors as a cost-effective and fault-tolerant alternative to physical instrumentation. Virtual sensors are data-driven software models that estimate or replicate the behavior of physical sensors when measurements are missing, unreliable, or unavailable [20]. These models are particularly beneficial in scenarios where sensor deployment is constrained by economic, safety, or environmental limitations. Early approaches primarily relied on linear regression and principal component analysis (PCA) for sensor approximation; however, these methods often fail to capture the nonlinear and complex dependencies inherent in real-world sensor data [21].

Robust data preprocessing is a critical prerequisite for accurate virtual sensor modeling. Sensor datasets frequently suffer from missing values, noise, and outliers, which can significantly degrade model performance if not properly addressed. Common imputation techniques, such as k-nearest neighbors (KNN) and model-based approaches, have been widely adopted, though their effectiveness depends on the nature and distribution of missing data [22,23]. For anomaly detection, Isolation Forest has emerged as a powerful unsupervised technique capable of identifying outliers in high-dimensional datasets, outperforming traditional statistical methods such as Z-score and interquartile range (IQR) in capturing multivariate relationships [24]. Nevertheless, existing preprocessing pipelines often lack domain-specific customization, limiting their robustness in complex and heterogeneous sensor networks.

To address data scarcity, synthetic data generation techniques have been extensively explored. Traditional approaches include Monte Carlo simulations [25], Gaussian mixture models [26], and copula-based modeling [27], which are particularly effective in capturing complex dependencies among multiple variables. Copula models enable the separation of marginal distributions from joint dependencies, making them well-suited for multivariate time-series sensor data. In addition, spatial interpolation techniques such as Inverse Distance Weighting (IDW) have been employed to estimate sensor values in geographically distributed environments [28]. Despite their effectiveness, purely statistical methods often fail to preserve underlying physical constraints and complex spatiotemporal dynamics, limiting their applicability in realistic sensor environments.

The validation of synthetic or virtual sensor data is typically performed using statistical similarity measures. Kernel Density Estimation (KDE) is widely used to assess distributional alignment [29], while divergence-based metrics such as Kullback–Leibler (KL) divergence provide a quantitative measure of similarity between real and generated data distributions [30,31]. These techniques are particularly useful in data fusion scenarios, where outputs from multiple generation methods can be combined to improve realism and generalization. However, conventional validation approaches primarily focus on marginal distributions and may not fully capture temporal dependencies or multivariate interactions without additional adaptations [32].

Recent advancements in deep generative models have introduced powerful data augmentation techniques for virtual sensor applications. Variational Autoencoders (VAE) enable the learning of latent representations while maintaining distributional regularization, making them suitable for generating statistically consistent synthetic data [33]. Similarly, Conditional Tabular Generative Adversarial Networks (CTGAN) are designed to handle mixed-type tabular data, effectively modeling both categorical and continuous features [34,35]. These generative approaches are often combined with deep sequence models such as Bidirectional Long Short-Term Memory (BiLSTM) and Bidirectional Gated Recurrent Units (BiGRU), which excel at capturing temporal dependencies in sensor data. However, most existing studies treat data generation, augmentation, and modeling as separate components, lacking a unified framework that integrates these processes into a cohesive pipeline [36].

Recent studies have also explored machine learning-assisted physical sensor systems for intelligent recognition tasks. For example, Dong et al. [37] developed a flexible dual-modal sensor for robotic electronic skin that simultaneously measures proximity distance and contact pressure during robotic grasping. Their system used an AlexNet neural network for target material and hardness recognition, achieving recognition rates of 93.49% for material classification and 92.22% for hardness classification. Although this work demonstrates the effectiveness of machine learning in physical multi-modal sensor-based recognition, its primary focus is on robotic tactile sensing and classification. In contrast, the present study focuses on virtual sensor generation for environmental monitoring, where the objective is to reconstruct and generate reliable sensor data under sparse, missing, or unreliable sensing conditions using statistical modeling, KL-divergence-based fusion, deep generative augmentation, and temporal prediction models.

To provide a more targeted comparison with recent sensor, virtual sensor, temporal prediction, and temporal generative studies, Table 1 summarizes the main focus, strengths, and limitations of representative works. Existing studies have made important contributions to virtual sensor modeling, spatial interpolation, dependency modeling, synthetic data generation, machine-learning-assisted physical sensing, temporal forecasting, and spatio-temporal learning. Recent Transformer-based time-series models and Temporal Fusion Transformers (TFTs) have shown strong capability in capturing long-range temporal dependencies and providing interpretable multi-horizon forecasting [38]. Similarly, Graph Neural Networks (GNNs) and spatio-temporal GNNs are effective for modeling spatial and temporal dependencies among connected sensor nodes or graph-structured time-series data [39,40]. In parallel, TimeGAN and diffusion-based time-series models have been widely explored for temporal synthetic data generation by learning sequential dependencies and complex data distributions [41,42].

However, most of these approaches focus on isolated components, such as temporal prediction, graph-based spatial dependency learning, synthetic sequence generation, interpolation, classification, or forecasting. Transformer-based models, TFTs, and GNNs primarily serve as prediction or spatio-temporal modeling approaches, whereas TimeGAN and diffusion-based models are mainly designed for temporal synthetic data generation. In contrast, the proposed framework integrates physical-constraint-based preprocessing, statistical virtual sensor generation, KL-divergence-based fusion, VAE/CTGAN-based augmentation, circular wind-direction encoding, and BiLSTM/BiGRU-based temporal prediction in a unified virtual sensing pipeline. This integration improves the reliability, distributional consistency, and predictive usefulness of generated virtual sensor data, although the framework still requires careful parameter selection and further validation when transferred to other sensor domains.

## 3. Proposed Methodology

**Figure 1:**
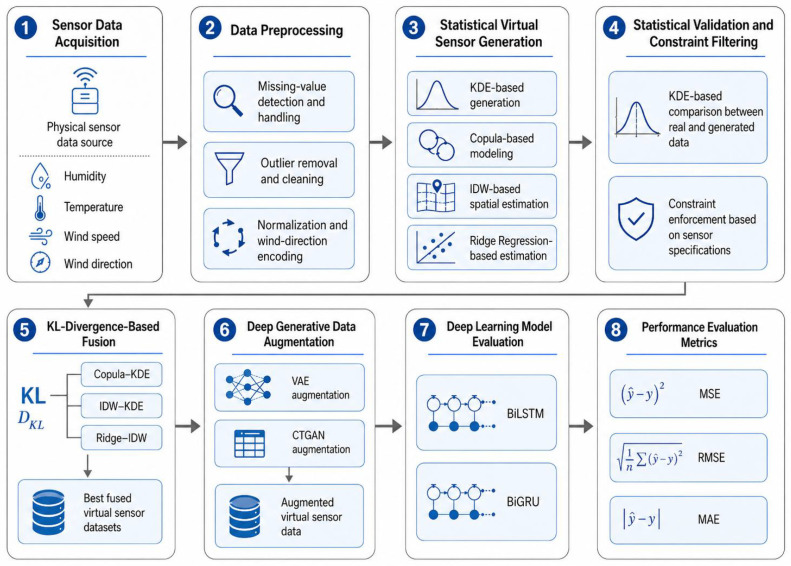
Proposed framework for virtual sensor development integrating preprocessing, statistical virtual sensor generation (KDE, Copula, IDW, and Ridge Regression), KDE-based validation, physical constraint filtering, KL-divergence-based data fusion, deep generative augmentation (VAE and CTGAN), and time-series modeling using BiLSTM/BiGRU for accurate prediction and reconstruction of sensor data.

The proposed methodology, illustrated in Figure 1, presents a structured and unified pipeline for virtual sensor development, leveraging both statistical modeling and deep learning techniques. The framework begins with data acquisition from physical sensors measuring key environmental parameters, including temperature, humidity, wind speed, and wind direction. In the initial stage, the raw sensor data undergo rigorous preprocessing to ensure data quality and reliability. This includes missing value detection and handling, as well as outlier removal using both threshold-based filtering and Isolation Forest, thereby eliminating noise and anomalous observations that could negatively impact downstream modeling.

Following preprocessing, virtual sensor data are generated using complementary simulation-based approaches. Specifically, KDE-based generation, Copula-based modeling, IDW-based spatial estimation, and Ridge Regression-based estimation are employed to generate complementary virtual sensor data by capturing distributional, dependency-based, spatial, and regression-based relationships among sensor variables. The generated synthetic datasets are subsequently subjected to statistical validation using Kernel Density Estimation (KDE) to assess distributional alignment with real data. In addition, constraint-based filtering is applied to enforce physically meaningful ranges based on sensor specifications, ensuring the realism of the generated data.

To further enhance data quality, the framework incorporates a KL-divergence-based fusion mechanism that adaptively combines outputs from different generation methods by prioritizing those with higher distributional similarity to the original data. This fusion process reduces individual model biases and produces a more robust and representative virtual dataset. The fused data is then augmented using deep generative models, including Variational Autoencoders (VAE) and Conditional Tabular Generative Adversarial Networks (CTGAN), to improve data diversity and generalization capability.

The enriched dataset is subsequently used to train advanced time-series learning models, namely Bidirectional Long Short-Term Memory (BiLSTM) and Bidirectional Gated Recurrent Unit (BiGRU), which are designed to capture complex temporal dependencies in sensor data. Finally, the performance of the proposed framework is evaluated using standard regression metrics, including Mean Squared Error (MSE), Root Mean Squared Error (RMSE), and Mean Absolute Error (MAE), demonstrating its effectiveness in accurately reconstructing and predicting sensor behavior.

### 3.1. Original Physical Sensor Data

The dataset utilized in this study is collected from multiple weather stations deployed across South Korea, comprising measurements from nine spatially distributed sensor nodes. Let the multivariate sensor dataset be represented as

(1) $$X=\{x_{t}\in R^{d}|t=1,2,\ldots ,T\}$$

where $x_{t}$ denotes the sensor observation at time *t*, *d* represents the number of measured environmental variables, and *T* is the total number of temporal observations. Each observation consists of meteorological attributes, including temperature, humidity, wind speed and wind direction angle, along with temporal information (timestamp) and spatial coordinates (longitude and latitude).

The data are recorded at regular 10-min intervals, providing high-resolution temporal coverage and enabling the modeling of fine-grained atmospheric dynamics. The inclusion of both spatial and temporal attributes allows the dataset to capture complex spatiotemporal dependencies inherent in environmental systems.

This dataset serves as the foundational input for the proposed virtual sensor generation framework. The availability of multi-location sensor measurements facilitates the modeling of microclimatic variations and supports the development of robust data-driven representations. Such characteristics are essential for applications including weather forecasting, environmental monitoring, and urban resilience planning, particularly in scenarios involving data sparsity or sensor failure.

A detailed description of the physical sensor variables, their data types, and corresponding environmental interpretations is provided in Table 2, highlighting the structure and granularity of the collected data.

### 3.2. Data Preprocessing and Analysis

#### 3.2.1. Physical Constraints and Outlier Handling

To ensure the realism and physical validity of both original and augmented sensor data, constraint-based filtering is applied. Let $x_{t}\in R^{d}$ denote a sensor observation at time *t*. Each feature is constrained within physically meaningful bounds:

(2) $$x_{t}^{\left(i\right)}\in \left[\alpha_{i},\beta_{i}\right],\;\forall i=1,\ldots ,d$$

where $\alpha_{i}$ and $\beta_{i}$ represent the lower and upper limits defined by sensor specifications and physical validity requirements. The physical constraints used for validating the sensor variables are summarized in Table 3. This step prevents implausible values from propagating into downstream modeling.

In addition, outlier detection is performed using Isolation Forest. The anomaly score for a sample x is defined as

(3) $$s\left(x\right)=2^{-\frac{E(h(x))}{c(n)}}$$

where E(h(x)) is the expected path length and c(n) is a normalization factor. Samples with lower path lengths, corresponding to higher anomaly scores, are identified as outliers and removed. This approach efficiently isolates anomalous points in high-dimensional sensor data.

#### 3.2.2. Circular Encoding of Wind Direction

Wind direction is a circular angular variable, and directly using its degree value as a linear numerical feature may introduce an artificial discontinuity at the $0^{\circ }/360^{\circ }$ boundary. For example, the wind directions of $1^{\circ }$ and $359^{\circ }$ are numerically far apart, although they are physically separated by only $2^{\circ }$. To preserve the periodic nature of wind direction, the original wind direction angle was transformed into two unit-circle components before model training and virtual sensor generation.

First, the wind direction angle θ in degrees was converted into radians:

(4) $$\theta_{\mathrm{rad}}=\frac{\pi }{180}\theta$$

Then, two circular components were computed as

(5) $$\mathrm{WindDirX}=\cos(\theta_{\mathrm{rad}})$$

(6) $$\mathrm{WindDirY}=\sin(\theta_{\mathrm{rad}})$$

where WindDirX and WindDirY represent the horizontal and vertical unit-circle components of wind direction, respectively. These two components were used as model inputs and outputs instead of directly modeling the wind direction angle in degrees. After prediction, the wind direction angle was reconstructed using the inverse tangent function:

(7) $$\hat{\theta }=\left(\frac{180}{\pi }a\tan2\left(\mathrm{WindDirY},\mathrm{WindDirX}\right)\right)\;\mathrm{mod}\;360$$

This encoding removes the artificial discontinuity at the $0^{\circ }/360^{\circ }$ boundary and enables the model to learn the physical closeness of circular wind directions. The reconstructed wind direction angle in degrees was used for visualization, interpretation, and performance evaluation.

### 3.3. Statistical Methods for Virtual Sensor Simulation

#### 3.3.1. Ridge Regression for Virtual Sensor Simulation

**Figure 2:**
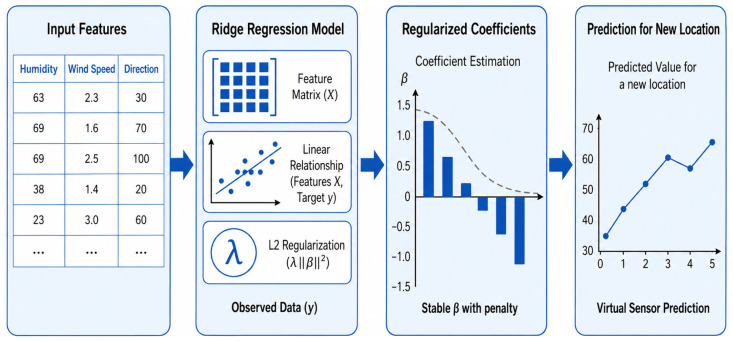
Ridge Regression flow for virtual sensor simulation.

Ridge Regression (Figure 2) is employed as a regularized regression-based method for virtual sensor simulation. Unlike ordinary least squares regression, Ridge Regression introduces an $L_{2}$ penalty term to reduce overfitting and improve numerical stability when sensor variables are noisy or highly correlated. Given the design matrix $X\in R^{n\times d}$ and target vector $y\in R^{n}$, the Ridge Regression objective is defined as

(8) $$\min_{\beta }{∥y-X\beta ∥}_{2}^{2}+\lambda {∥\beta ∥}_{2}^{2}$$

where β denotes the regression coefficient vector and λ is the regularization parameter that controls the strength of the $L_{2}$ penalty. A larger value of λ increases regularization and produces smoother coefficient estimates, while a smaller value allows the model to fit the training data more closely.

The closed-form solution of Ridge Regression is given by:

(9) $$\hat{\beta }={\left(X^{T}X+\lambda I\right)}^{-1}X^{T}y$$

where I is the identity matrix. For a new sensor observation $x_{*}$, the corresponding virtual sensor estimate is computed as

(10) $${\hat{y}}_{*}=x_{*}^{T}\hat{\beta }$$

In this study, Ridge Regression is used as one of the statistical virtual sensor generation components and is combined with IDW in the KL-Ridge-IDW fusion configuration. The regularization term helps produce stable virtual sensor estimates under noisy and correlated environmental sensor conditions, while the subsequent KL-divergence-based fusion step selects and combines generated outputs according to their distributional similarity with the original physical sensor data.

#### 3.3.2. Inverse Distance Weighting (IDW)

**Figure 3:**
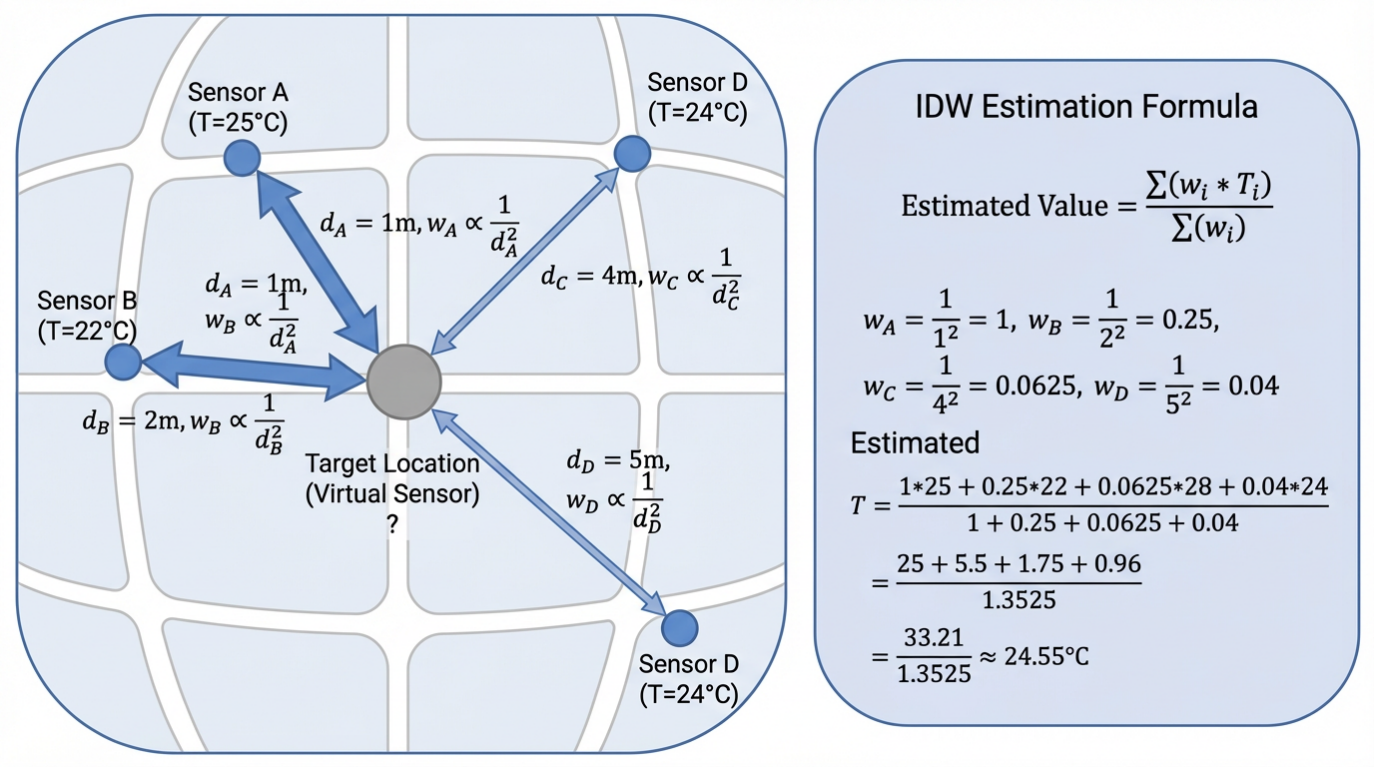
Inverse Distance Weighting workflow for virtual sensor estimation.

Inverse Distance Weighting (IDW), shown in Figure 3, is employed for spatial interpolation. The estimated value at an unknown location $s_{0}$ is computed as

(11) $$\hat{x}\left(s_{0}\right)=\frac{\sum_{i=1}^{N}w_{i}x\left(s_{i}\right)}{\sum_{i=1}^{N}w_{i}}$$

where weights are defined as

(12) $$w_{i}=\frac{1}{∥s_{0}-s_{i}{∥}^{p}}$$

Here, *p* controls the influence of distance. This formulation ensures that closer observations have a higher impact, preserving spatial consistency.

**Figure 4:**
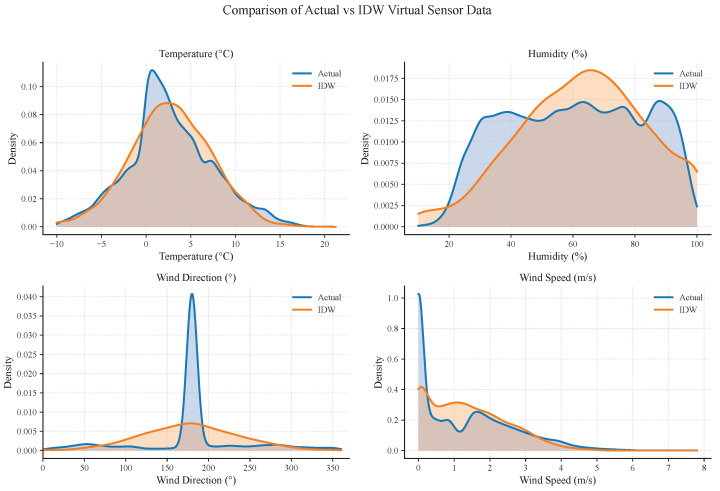
Density comparison between real sensor data and IDW-generated virtual sensor data for temperature, humidity, wind direction, and wind speed.

The density plots in Figure 4 demonstrate that IDW effectively captures local spatial patterns, particularly for temperature and humidity. However, it exhibits limitations in modeling directional or highly skewed variables such as wind direction and wind speed.

#### 3.3.3. Kernel Density Estimation (KDE)

Kernel Density Estimation (KDE) is used to estimate the probability density function of sensor variables without assuming a parametric distribution. Given observations ${\left\{x_{i}\right\}}_{i=1}^{n}$, KDE is defined as

(13) $$\hat{f}\left(x\right)=\frac{1}{nh}\sum_{i=1}^{n}K\left(\frac{x-x_{i}}{h}\right)$$

where K(·) is a kernel function (e.g., Gaussian) and *h* is the bandwidth controlling smoothness.

**Figure 5:**
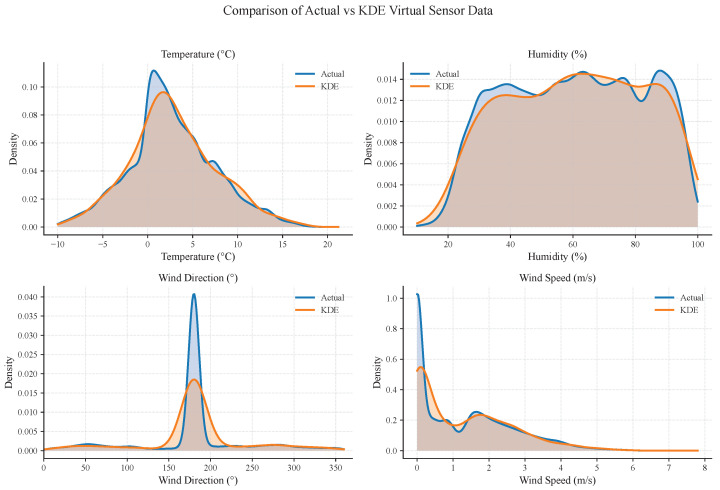
Density comparison between real sensor data and KDE-generated virtual sensor data for temperature, humidity, wind direction, and wind speed.

As illustrated in Figure 5, KDE effectively preserves the overall distributional structure of the original data. It produces smooth approximations of temperature and humidity distributions while slightly smoothing peaks in wind-related features.

#### 3.3.4. Copula-Based Virtual Sensor Data Generation

Copula-based modeling enables joint distribution learning by separating marginal distributions and dependency structure:

(14) $$F\left(x_{1},\ldots ,x_{d}\right)=C\left(F_{1}\left(x_{1}\right),\ldots ,F_{d}\left(x_{d}\right)\right)$$

Synthetic samples are generated via:

(15) $$u∼C\left(u_{1},\ldots ,u_{d}\right),\;x_{i}=F_{i}^{-1}\left(u_{i}\right)$$

This approach preserves both marginal behavior and inter-variable dependencies.

**Figure 6:**
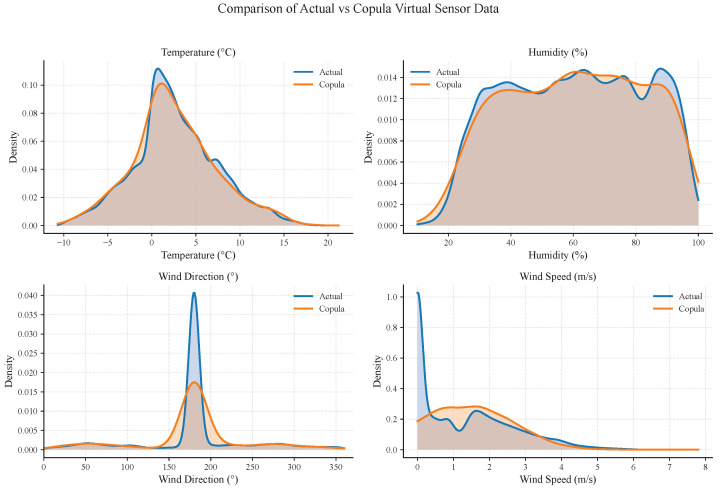
Density comparison between real sensor data and Copula-generated virtual sensor data.

As shown in Figure 6, copula-based generation closely aligns with real data distributions, particularly for temperature and humidity, while maintaining realistic correlations among variables.

#### 3.3.5. KL-Divergence-Based Data Fusion

To combine multiple synthetic datasets, Kullback–Leibler divergence is used:

(16) $$D_{KL}\left(P\|Q_{k}\right)=\sum_{x}P\left(x\right)\log\frac{P(x)}{Q_{k}\left(x\right)}$$

Fusion weights are computed as

(17) $$w_{k}=\frac{1/D_{KL}\left(P\|Q_{k}\right)}{\sum_{j}1/D_{KL}\left(P\|Q_{j}\right)}$$

The fused dataset is obtained by:

(18) $$X_{fusion}=\sum_{k}w_{k}X_{k}$$

**Figure 7:**
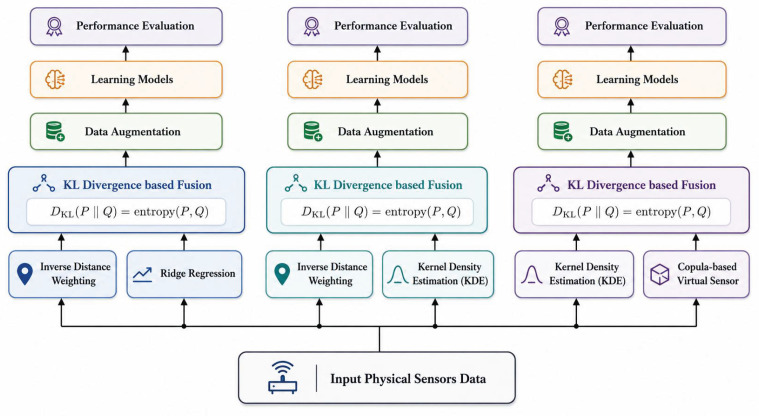
KL-divergence-based fusion of virtual sensor data generated from different statistical methods. The fused data are validated within the defined physical ranges before augmentation and downstream model training.

Figure 7 illustrates the fusion architecture combining different statistical models.

#### 3.3.6. Hybrid Fusion Configurations

Three fusion strategies are explored:

- IDW + Ridge Regression
- IDW + KDE
- Copula + KDE

The objective is:

(19) $$\min_{w_{k}}\sum_{k}w_{k}D_{KL}\left(P\|Q_{k}\right)$$

**Figure 8:**
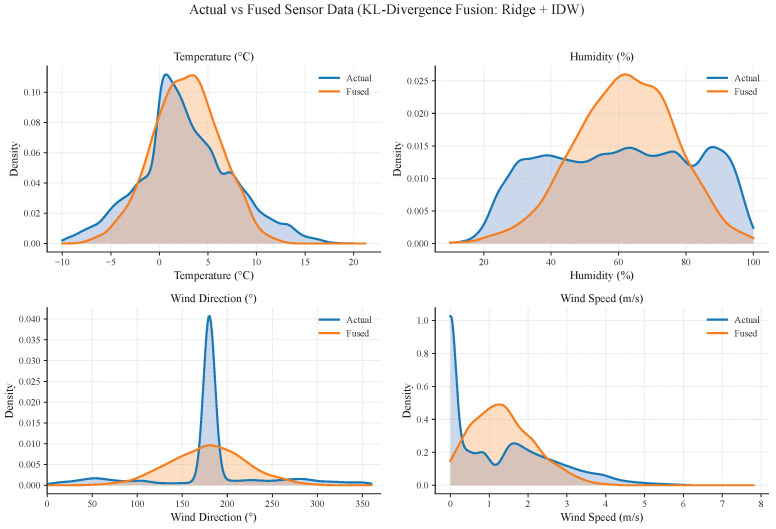
Density comparison of the fused IDW–Ridge virtual sensor data.

**Figure 9:**
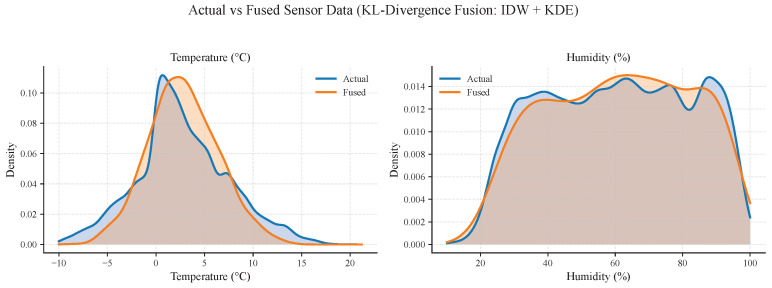

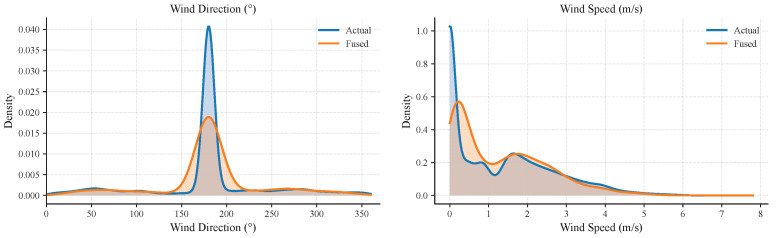
Density comparison of the fused IDW–KDE virtual sensor data.

**Figure 10:**
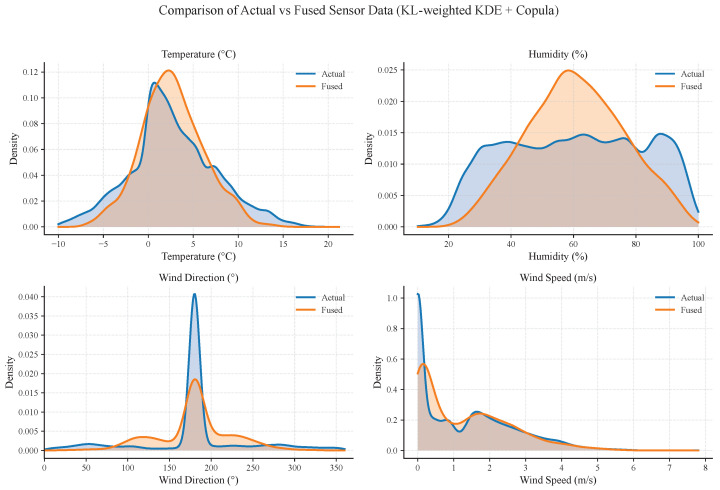
Density comparison of the fused Copula–KDE virtual sensor data.

Figure 8, Figure 9 and Figure 10 show that fusion improves distributional alignment, with Copula + KDE providing the best overall performance by combining dependency modeling and smooth density estimation.

### 3.4. Enhanced Virtual Sensor Data Augmentation Using VAE and CTGAN

To enhance the diversity and robustness of the initially generated virtual sensor data, this study incorporates advanced data augmentation using deep generative models. Let the statistically generated virtual sensor dataset from the previous stage be denoted as

(20) $$X_{s}=\left\{x_{i}\in R^{d}|i=1,2,\ldots ,N\right\}$$

where $X_{s}$ represents synthetic data obtained from statistical methods such as IDW, KDE, Ridge Regression, and Copula-based modeling. The objective of augmentation is to learn the underlying data distribution $p_{data}\left(x\right)$ and generate additional samples:

(21) $$\tilde{x}∼p_{\theta }\left(x\right)\approx p_{data}\left(x\right)$$

such that the augmented dataset preserves both statistical and structural properties of the original data.

To achieve this, two complementary deep generative frameworks are employed: Variational Autoencoder (VAE) and Conditional Tabular Generative Adversarial Network (CTGAN). These models aim to approximate the true data distribution by learning a mapping from a latent space z∼p(z) to the data space:

(22) $$x=G_{\theta }\left(z\right)$$

where $G_{\theta }\left(\cdot \right)$ denotes the generative function parameterized by neural networks.

The augmented dataset is constructed by combining original synthetic samples with generated samples:

(23) $$X_{aug}=X_{s}\cup \tilde{X}$$

where $\tilde{X}$ represents newly generated samples from deep generative models.

**Figure 11:**
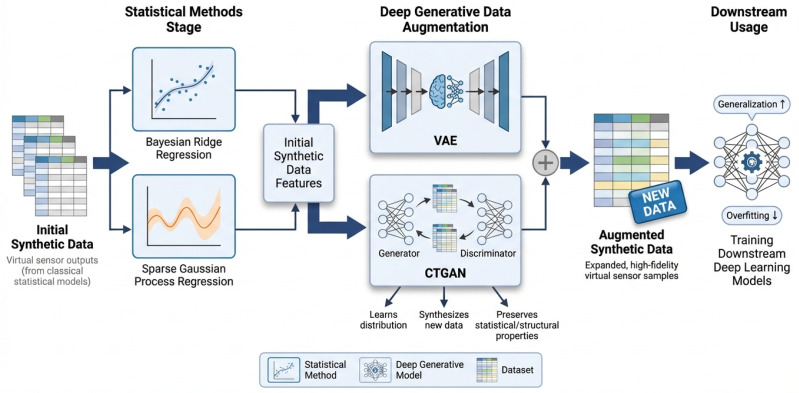
Augmentation of the initial data generated using statistical models, followed by deep generative modeling (VAE and CTGAN) to produce high-fidelity synthetic sensor data for downstream learning.

As illustrated in Figure 11, the statistical outputs are first transformed into initial synthetic features, which are then passed through VAE and CTGAN models for distribution learning and sample generation. VAE focuses on learning a compact latent representation and reconstructing statistically consistent samples, while CTGAN emphasizes capturing complex tabular dependencies and generating diverse samples conditioned on feature distributions.

This augmentation process enhances data diversity and improves the generalization capability of downstream models by reducing overfitting:

(24) $$R_{gen}\propto \frac{1}{|X_{aug}|}$$

where $R_{gen}$ denotes generalization error, which decreases as the dataset size and diversity increase.

Consequently, the augmented dataset provides a more comprehensive representation of the underlying sensor dynamics, enabling improved learning of temporal and statistical patterns in subsequent deep learning stages.

#### 3.4.1. Variational Autoencoder (VAE)

**Figure 12:**
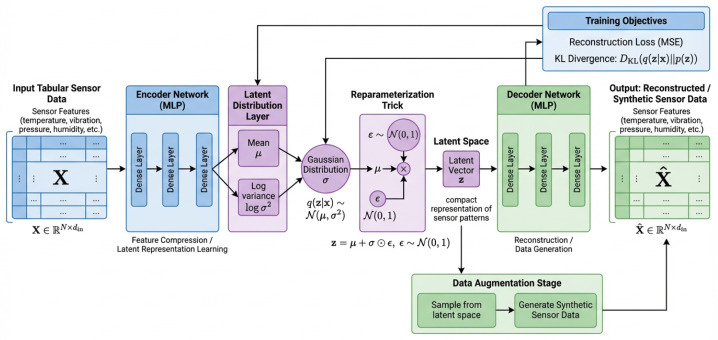
Architecture of the Variational Autoencoder (VAE) model, illustrating the encoder, latent space sampling, and decoder used for generating augmented sensor data.

The Variational Autoencoder (VAE) is a probabilistic generative model that learns a latent representation of data and generates new samples consistent with the learned distribution. As shown in Figure 12, the model consists of an encoder, a latent sampling mechanism, and a decoder.

The encoder maps input data $x\in R^{n}$ to a latent Gaussian distribution:

(25) $$q_{\phi }\left(z|x\right)=N\left(z;\mu \left(x\right),\sigma^{2}\left(x\right)\right)$$

where

(26) $$\mu =f_{\mu }\left(x\right),\;\log\sigma^{2}=f_{\sigma }\left(x\right)$$

To enable gradient-based optimization, the reparameterization trick is applied:

(27) z=μ+σ⊙ϵ,ϵ∼N(0,I)

The decoder reconstructs the input:

(28) $$\hat{x}∼p_{\theta }\left(x|z\right)$$

The model is trained by maximizing the Evidence Lower Bound (ELBO):

(29) $$L\left(\theta ,\phi ;x\right)=E_{q_{\phi }\left(z|x\right)}\left[\log p_{\theta }\left(x|z\right)\right]-D_{\mathrm{KL}}\left(q_{\phi }\left(z|x\right)∥p\left(z\right)\right)$$

where p(z)=N(0,I) is the prior. The first term ensures reconstruction fidelity, while the KL divergence regularizes the latent space.

By sampling z∼p(z) and decoding, new synthetic samples are generated:

(30) $$\tilde{x}=G_{\theta }\left(z\right)$$

This enables the generation of statistically consistent virtual sensor data, improving dataset diversity and enhancing generalization in downstream learning tasks.

#### 3.4.2. Conditional Tabular Generative Adversarial Network (CTGAN)

**Figure 13:**
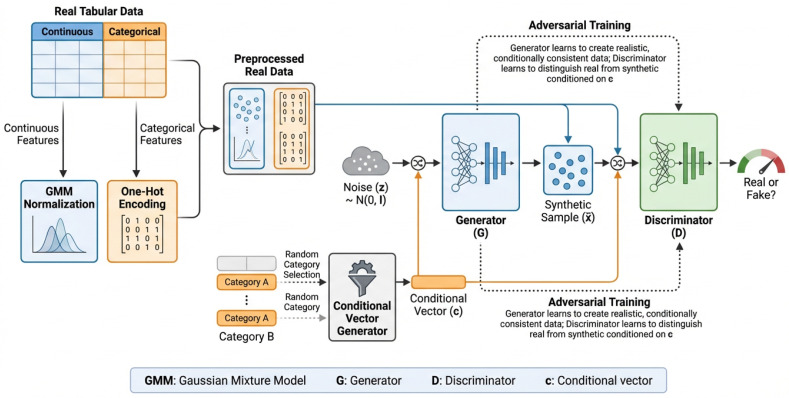
Architecture of the CTGAN model used for generating augmented sensor data.

The Conditional Tabular Generative Adversarial Network (CTGAN) is designed for generating synthetic tabular data with mixed continuous and categorical features. As illustrated in Figure 13, CTGAN consists of a generator *G*, discriminator *D*, and a conditioning mechanism for controlled data synthesis.

Let the real dataset be $X={\left\{x_{i}\right\}}_{i=1}^{N}$, consisting of continuous and categorical features. Continuous variables are transformed using Gaussian Mixture Models (GMM):

(31) $$p\left(x\right)=\sum_{k=1}^{K}\pi_{k}N\left(x|\mu_{k},\Sigma_{k}\right)$$

while categorical variables are encoded using one-hot representations.

To enable conditional generation, a condition vector *c* is sampled from the categorical feature space and concatenated with noise:

(32) $$z∼N\left(0,I\right),\;\tilde{x}=G\left(z,c\right)$$

The discriminator receives both data and condition:

(33) D(x,c)→[0,1]

The adversarial objective is defined as

(34) $$\min_{G}\max_{D}\;E_{x∼p_{\mathrm{data}}}\left[\log D\left(x,c\right)\right]+E_{z∼p(z)}\left[\log\left(1-D\left(G\left(z,c\right),c\right)\right)\right]$$

This formulation ensures that *G* learns the conditional distribution p(x|c), while *D* enforces realism.

The conditioning mechanism improves learning in imbalanced datasets and preserves feature dependencies. As a result, CTGAN generates diverse synthetic samples:

(35) $$\tilde{X}={\left\{G\left(z_{i},c_{i}\right)\right\}}_{i=1}^{M}$$

which expand the dataset and enhance representation capacity.

Compared to VAE, CTGAN emphasizes diversity and mode coverage, making it effective for capturing complex tabular distributions, although it may introduce slight distributional variance.

#### 3.4.3. Comparison of VAE and CTGAN

To quantitatively and qualitatively evaluate the effectiveness of deep generative augmentation methods, this section compares Variational Autoencoder (VAE) and Conditional Tabular GAN (CTGAN) across three fused data configurations: (i) Copula + KDE, (ii) IDW + KDE, and (iii) Ridge + IDW. Let $X_{fusion}$ denote the fused dataset and $X_{aug}^{VAE}$, $X_{aug}^{CTGAN}$ represent augmented datasets:

(36) $$X_{aug}^{VAE}=X_{fusion}\cup {\tilde{X}}_{VAE},\;X_{aug}^{CTGAN}=X_{fusion}\cup {\tilde{X}}_{CTGAN}$$

The comparison evaluates distributional similarity and structural preservation using PCA projections and KDE-based density estimation. PCA transforms data into a lower-dimensional space:

(37) Z=XW

where W contains principal components maximizing variance.

**a.**  **Fused Copula & KDE Weighted Virtual Sensor Data Augmentation Analysis**

**Figure 14:**
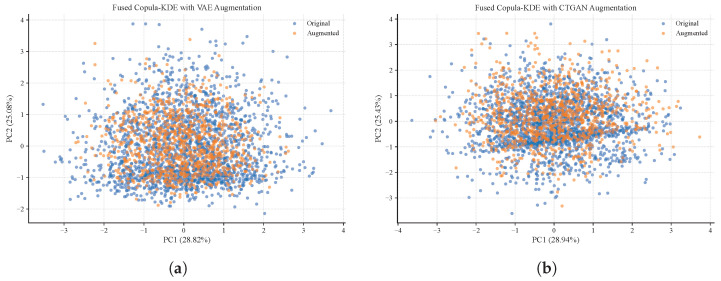
Fused Copula & KDE weighted virtual sensor data augmentation analysis using PCA visualization: (**a**) VAE-augmented data and (**b**) CTGAN-augmented data.

For the Copula-KDE fused dataset, both VAE and CTGAN effectively preserve the global data structure, as observed in the PCA visualization (Figure 14). VAE-generated samples exhibit compact clustering around the principal data manifold, indicating strong adherence to the original distribution. In contrast, CTGAN produces a broader spread:

(38) $$\mathrm{Var}\left(X_{aug}^{CTGAN}\right)>\mathrm{Var}\left(X_{aug}^{VAE}\right)$$

suggesting increased variability.

**Figure 15:**
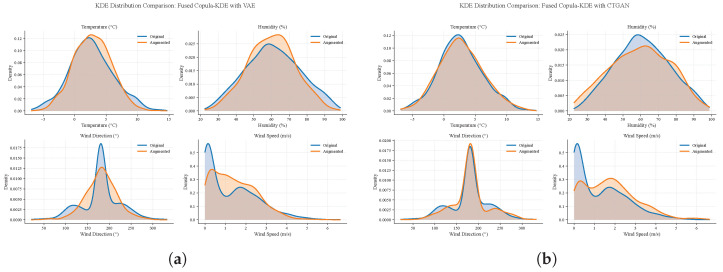
KDE-based distribution plots of feature-wise similarity for fused Copula & KDE weighted virtual sensor data: (**a**) VAE-augmented data and (**b**) CTGAN-augmented data.

The KDE-based distributions (Figure 15) further confirm that VAE maintains closer alignment with the empirical distribution:

(39) $$D_{KL}\left(P\|Q_{VAE}\right)<D_{KL}\left(P\|Q_{CTGAN}\right)$$

particularly for temperature and humidity. CTGAN, however, captures heavier tails in wind-related features, indicating improved representation of rare events but with slight deviation in mean alignment.

**b.**  **Fused KL-Based IDW & KDE Virtual Sensor Data Augmentation Analysis**

**Figure 16:**
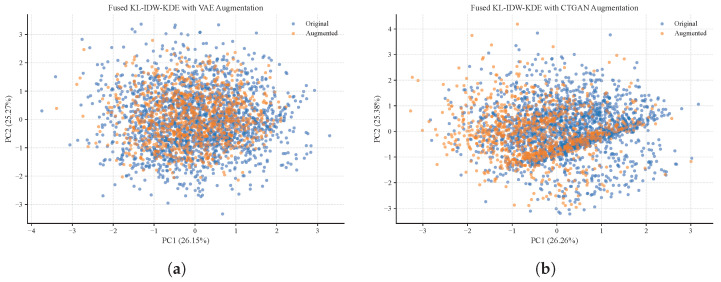
Fused KL-based IDW & KDE virtual sensor data augmentation analysis using PCA visualization: (**a**) VAE-augmented data and (**b**) CTGAN-augmented data.

In the KL-IDW-KDE configuration, PCA results (Figure 16) show that VAE-generated samples remain tightly concentrated around the original data distribution:

(40) $${∥X_{aug}^{VAE}-X_{real}∥}_{2}\ll {∥X_{aug}^{CTGAN}-X_{real}∥}_{2}$$

indicating higher statistical fidelity. CTGAN again introduces greater dispersion, reflecting enhanced diversity.

**Figure 17:**
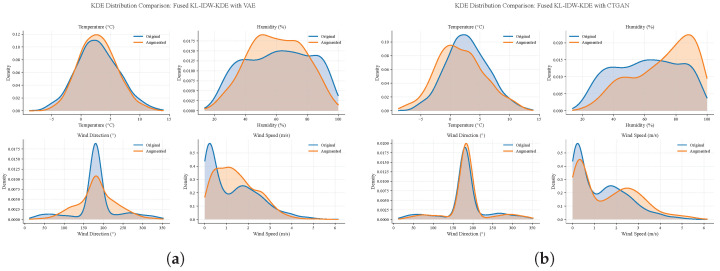
Density distribution analysis of fused KL-based IDW & KDE virtual sensor data augmentation: (**a**) VAE-augmented data and (**b**) CTGAN-augmented data.

Density plots (Figure 17) highlight that VAE preserves central tendencies and distribution peaks more accurately, while CTGAN produces broader distributions:

(41) $${\hat{f}}_{CTGAN}\left(x\right)={\hat{f}}_{real}\left(x\right)+\delta \left(x\right),\;\left|\delta \left(x\right)\right|>0$$

particularly in humidity and wind speed. This demonstrates a trade-off between fidelity and diversity.

**c.**  **Fused KL-Based Ridge & IDW Virtual Sensor Data Augmentation Analysis**

**Figure 18:**
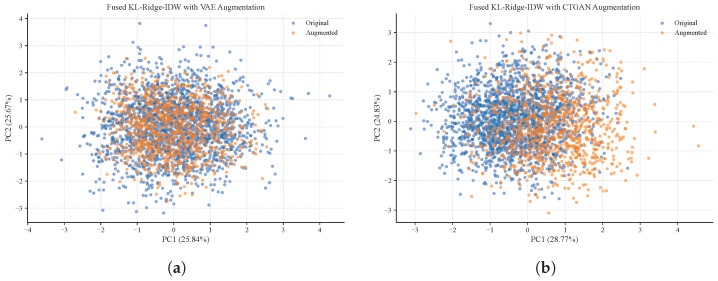
Fused KL-based Ridge & IDW virtual sensor data augmentation analysis using PCA visualization: (**a**) VAE-augmented data and (**b**) CTGAN-augmented data.

For the Ridge-IDW fused dataset, PCA results (Figure 18) show that VAE maintains a dense and structured representation within the original data manifold:

(42) $$\mathrm{Cov}\left(X_{aug}^{VAE}\right)\approx \mathrm{Cov}\left(X_{real}\right)$$

while CTGAN exhibits larger dispersion, indicating exploratory sampling behavior.

**Figure 19:**
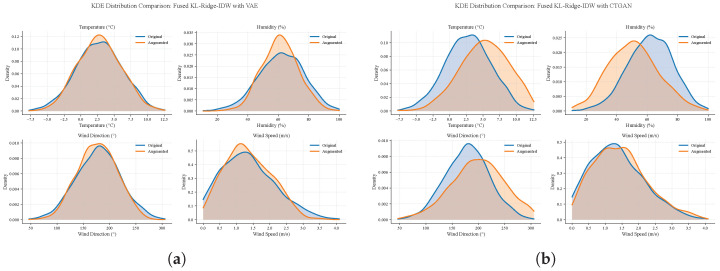
Density distribution analysis of fused KL-based Ridge & IDW virtual sensor data augmentation: (**a**) VAE-augmented data and (**b**) CTGAN-augmented data.

The density distributions (Figure 19) further validate that VAE closely preserves feature-wise distributions:

(43) $${\hat{f}}_{VAE}\left(x\right)\approx {\hat{f}}_{real}\left(x\right)$$

whereas CTGAN introduces shifts toward extreme values, particularly in humidity and temperature, indicating higher variance but potential distributional drift.

**Overall Comparison**

Across all fusion configurations, VAE consistently demonstrates superior performance in preserving statistical fidelity and structural integrity, as evidenced by:

(44) $$D_{KL}\left(P\|Q_{VAE}\right)<D_{KL}\left(P\|Q_{CTGAN}\right)$$

and tighter clustering in PCA space. In contrast, CTGAN provides higher diversity:

(45) $$\mathrm{Var}\left(X_{aug}^{CTGAN}\right)>\mathrm{Var}\left(X_{aug}^{VAE}\right)$$

which is beneficial for capturing rare patterns but may introduce distributional deviations.

Therefore, VAE is more suitable for applications requiring high accuracy and distributional consistency, while CTGAN is advantageous in scenarios where diversity and exploratory data generation are prioritized.

### 3.5. Deep Learning Evaluation

#### 3.5.1. BiLSTM

Bidirectional Long Short-Term Memory (BiLSTM) models temporal dependencies by processing the input sequence in both forward and backward directions. Given an input sequence ${\left\{x_{t}\right\}}_{t=1}^{T}$:

(46) $$\vec{h_{t}}=\mathrm{LSTM}\left(x_{t},{\vec{h}}_{t-1}\right)$$

(47) $$\overset{\leftarrow }{h_{t}}=\mathrm{LSTM}\left(x_{t},{\overset{\leftarrow }{h}}_{t+1}\right)$$

The final hidden representation is obtained by concatenation:

(48) $$h_{t}=\left[\vec{h_{t}};\overset{\leftarrow }{h_{t}}\right]$$

The output prediction is computed via:

(49) $${\hat{y}}_{t}=\sigma \left(W_{o}h_{t}+b_{o}\right)$$

Dropout regularization is applied during training:

(50) $$h_{t}^{drop}=\mathrm{Dropout}\left(h_{t},p\right)$$

where *p* is the dropout probability.

#### 3.5.2. BiGRU

Bidirectional Gated Recurrent Unit (BiGRU) is a computationally efficient alternative to BiLSTM, utilizing gating mechanisms to control information flow. For each time step:

**Update gate:**

(51) $$z_{t}=\sigma \left(W_{z}x_{t}+U_{z}h_{t-1}+b_{z}\right)$$

**Reset gate:**

(52) $$r_{t}=\sigma \left(W_{r}x_{t}+U_{r}h_{t-1}+b_{r}\right)$$

**Candidate state:**

(53) $${\tilde{h}}_{t}=\tan h\left(W_{h}x_{t}+U_{h}\left(r_{t}⊙h_{t-1}\right)+b_{h}\right)$$

**Hidden state update:**

(54) $$h_{t}=\left(1-z_{t}\right)⊙h_{t-1}+z_{t}⊙{\tilde{h}}_{t}$$

BiGRU extends this formulation bidirectionally:

(55) $$\vec{h_{t}}=\mathrm{GRU}\left(x_{t},{\vec{h}}_{t-1}\right),\;\overset{\leftarrow }{h_{t}}=\mathrm{GRU}\left(x_{t},{\overset{\leftarrow }{h}}_{t+1}\right)$$

(56) $$h_{t}=\left[\vec{h_{t}};\overset{\leftarrow }{h_{t}}\right]$$

The final prediction is:

(57) $${\hat{y}}_{t}=\sigma \left(W_{o}h_{t}+b_{o}\right)$$

**Notation:** $x_{t}$ denotes input at time *t*, $h_{t}$ hidden state, σ sigmoid function, tanh hyperbolic tangent, and ⊙ element-wise multiplication.

### 3.6. Experimental Setup and Model Configuration

To enhance the reproducibility and transparency of the proposed virtual sensor framework, this section summarizes the experimental computing environment, deep generative model settings, and temporal prediction model configurations. All experiments were conducted using the same preprocessing workflow, physical validity constraints, and data-splitting strategy to ensure a fair comparison across the generated, fused, and augmented virtual sensor datasets. In addition, wind direction was represented using WindDirX and WindDirY components during modeling to preserve its circular nature, and the final wind direction angle was reconstructed for evaluation and interpretation.

The computational environment used for training, augmentation, prediction, and evaluation is summarized in Table 4. The experiments were implemented in Python using standard data processing, machine learning, deep learning, and visualization libraries.

The key hyperparameters of the VAE and CTGAN models used for deep generative augmentation are presented in Table 5. These models were trained on the preprocessed and fused virtual sensor datasets to generate additional valid samples while preserving the statistical properties of the original sensor data.

The temporal prediction models used to evaluate the quality of the generated and augmented virtual sensor data are summarized in Table 6. Both BiLSTM and BiGRU were configured with the same input sequence length, optimizer, learning rate, loss function, batch size, and train-test split to ensure a consistent comparison between the two sequence-learning models.

To further support reproducibility, all experiments followed the same preprocessing and evaluation workflow. Missing or invalid values were removed, physical validity constraints were applied, numerical variables were scaled before model training, and generated samples were inverse-transformed and filtered using the same physical constraints before evaluation. The same 80% training and 20% testing split, sequence length, model settings, and evaluation metrics were used across all compared datasets and models.

### 3.7. Computational Complexity and Scalability Analysis

The proposed framework includes preprocessing, virtual sensor generation, KL-divergence-based fusion, VAE/CTGAN-based augmentation, and BiLSTM/BiGRU-based temporal prediction. The preprocessing operations, including missing-value handling, physical validity checking, normalization, and wind-direction transformation, scale linearly with the number of sensor records. In contrast, statistical fusion, deep generative augmentation, and temporal prediction are more computationally demanding because they involve density estimation, distributional comparison, and iterative neural network training.

Table 7 summarizes the computational characteristics of each major stage. Overall, the most time-consuming components are VAE/CTGAN augmentation and BiLSTM/BiGRU training, while memory usage mainly depends on the number of records, feature dimensions, generated samples, and batch size. In this study, all experiments were completed on a workstation with 96 GB RAM and an Intel Core i9-12900K processor, indicating that the proposed framework is feasible for medium-scale environmental sensor datasets.

To further quantify computational efficiency, training time, total inference time, single-sample inference time, and memory consumption were measured for the main augmentation and temporal prediction components. The measurements were obtained on the same workstation described in Table 4. For the prediction models, the reported training and prediction times represent the total time required to model all evaluated sensor variables, including temperature, humidity, wind speed, and the two-component wind-direction representation. The single-inference time denotes the average time required to predict one test sample after model training. These measurements provide a practical estimate of the runtime and memory requirements of the proposed framework.

In Table 8, *E* denotes the number of training epochs, *n* is the number of training samples, $n_{\mathrm{test}}$ is the number of test samples, *T* is the input sequence length, *h* is the number of hidden units, and *d* is the feature dimension. For recurrent prediction models, the training complexity is expressed as $O(EnTh^{2})$, while the prediction or inference complexity is expressed as $O(n_{\mathrm{test}}Th^{2})$. Sequence construction has linear complexity O(nT) and is therefore less computationally demanding than recurrent model training.

For larger sensor networks, scalability can be improved through parallel processing across sensor variables, monitoring locations, or time windows. The framework can also be extended using mini-batch training, incremental preprocessing, distributed storage, and location-wise model training. However, real-time deployment over very large multi-location sensor networks may require further optimization, such as model compression, efficient fusion updates, and streaming-based implementation.

## 4. Experimental Results and Analysis

Table 9 presents the baseline prediction performance of the BiLSTM and BiGRU models using the original physical sensor data. For temperature prediction, BiLSTM achieves lower errors than BiGRU, with MAE, MSE, and RMSE values of 1.4488, 4.1438, and 2.0356, respectively, compared with 1.6306, 4.9041, and 2.2145 for BiGRU. This indicates that BiLSTM provides better temperature modeling on the original sensor data. For humidity, both models achieve substantially lower errors, with BiLSTM producing slightly better performance than BiGRU. Specifically, BiLSTM obtains MAE, MSE, and RMSE values of 5.2799, 70.6272, and 8.4040, respectively, while BiGRU obtains corresponding values of 5.3701, 73.6730, and 8.5833. For wind direction, both models show very close performance, although BiGRU slightly improves the MAE from 0.5430 to 0.5353. For wind speed, BiLSTM provides a marginally lower MAE of 0.7449 compared with 0.7707 for BiGRU. These results show that BiLSTM is more effective for temperature, humidity, and wind speed, whereas BiGRU remains slightly more suitable for wind direction modeling.

Table 10 reports the performance of the fused Copula–KDE virtual sensor data and its CTGAN- and VAE-augmented variants. For the fused Copula–KDE baseline, BiLSTM and BiGRU achieve similar humidity performance, with MAE values of 13.1610 and 13.1704, respectively. For the Fused-CTGAN data, the humidity MAE values are slightly higher, reaching 13.7330 and 13.7730 for BiLSTM and BiGRU, respectively. The best humidity performance in this group is obtained using the Fused-VAE data, where BiLSTM and BiGRU reduce the MAE to 10.9138 and 10.6668, respectively. For temperature, both models show similar performance, with MAE values of 2.7053 and 2.7108 for BiLSTM and BiGRU, respectively. When CTGAN augmentation is applied, temperature errors increase to MAE values of 3.0941 and 3.0787, indicating that CTGAN introduces additional variability that does not benefit temperature prediction. In contrast, VAE augmentation produces the lowest temperature errors, reducing MAE to 2.2690 for both BiLSTM and BiGRU and decreasing RMSE to approximately 2.82. VAE also provides the best wind direction performance, achieving MAE values of 0.1628 and 0.1594 for BiLSTM and BiGRU, respectively, compared with 0.2795 and 0.2794 for the fused baseline. For wind speed, VAE reduces the MAE from approximately 1.09 in the fused baseline to about 0.88. These results demonstrate that VAE augmentation improves the predictive quality of fused Copula–KDE virtual sensor data, particularly for humidity, temperature, wind direction, and wind speed.

Table 11 presents the results for the KL-IDW–KDE fused virtual sensor dataset. In the fused baseline, BiLSTM and BiGRU show comparable temperature performance, with MAE values of 3.0261 and 3.0364, respectively. For humidity prediction, both models also show similar performance, with MAE values of 18.3344 and 18.3100 for BiLSTM and BiGRU, respectively. CTGAN augmentation does not consistently improve performance; for example, temperature MAE increases to 3.2386 and 3.2479 for BiLSTM and BiGRU, respectively, while wind speed MAE increases from approximately 1.02 to about 1.20. However, CTGAN provides some improvement for humidity, reducing the MAE to 16.5029 and 16.3493 for BiLSTM and BiGRU, respectively. In contrast, VAE augmentation provides clearer improvements across most variables. Temperature MAE decreases to 2.4581 and 2.4637, humidity MAE decreases to 14.9861 and 14.9666, and wind speed MAE decreases to 0.7732 and 0.7688 for BiLSTM and BiGRU, respectively. Wind direction prediction also improves, with MAE reduced from 0.4760 and 0.4460 in the fused baseline to 0.3217 and 0.3293 after VAE augmentation. These findings confirm that VAE is more effective than CTGAN for preserving the distributional structure of the KL-IDW–KDE fused data and improving downstream prediction accuracy.

Table 12 summarizes the performance of the KL-Ridge–IDW fused virtual sensor data. For the fused Ridge–IDW baseline, BiLSTM and BiGRU achieve similar humidity performance, with MAE values of 11.8917 and 11.8641, respectively. For the CTGAN-augmented variant, the humidity MAE values are 14.5449 and 14.5747 for BiLSTM and BiGRU, respectively. VAE augmentation provides the strongest humidity performance in this group, with BiLSTM achieving the lowest MAE of 10.2490 and BiGRU obtaining a comparable MAE of 10.6282. In addition, VAE augmentation provides the most consistent improvement across the wind-related variables. In particular, wind speed MAE decreases to 0.5896 and 0.5911 for BiLSTM and BiGRU, respectively, which are the lowest wind speed errors among the KL-Ridge–IDW variants. VAE also achieves the lowest wind direction MAE for BiLSTM, reducing the value to 0.1665, while BiGRU obtains a comparable value of 0.1739. These results indicate that the KL-Ridge–IDW configuration benefits strongly from VAE augmentation, especially for humidity and wind-related variables.

Table 13 presents the results for the standalone KDE-based virtual sensor dataset and its augmented variants. The KDE baseline produces higher prediction errors than the fusion-based methods. For example, temperature MAE reaches 3.8630 and 3.8839 for BiLSTM and BiGRU, respectively. For humidity prediction, the KDE baseline produces MAE values of 18.8129 and 18.8980 for BiLSTM and BiGRU, respectively. CTGAN does not improve humidity prediction for the standalone KDE data, increasing the humidity MAE to 22.2177 and 22.3357 for BiLSTM and BiGRU, respectively. In contrast, VAE provides the best humidity performance among the KDE variants, reducing MAE to 13.6639 and 13.7080 for BiLSTM and BiGRU, respectively. VAE also improves temperature, wind direction, and wind speed prediction compared with the KDE baseline, reducing temperature MAE to 3.6626 and 3.6764, wind direction MAE to 0.3844 and 0.3875, and wind speed MAE to 0.8657 and 0.8502 for BiLSTM and BiGRU, respectively. Although VAE improves the standalone KDE results, the errors remain higher than those obtained using fused virtual sensor data. This confirms that KL-divergence-based fusion is more effective than standalone KDE generation for producing reliable virtual sensor data.

### 4.1. Comparison

This section compares the MAE values of BiLSTM and BiGRU across the physical sensor data and all virtual sensor generation strategies. Since MAE directly represents the average prediction error, lower MAE values indicate higher predictive accuracy and better virtual sensor reliability.

**Figure 20:**
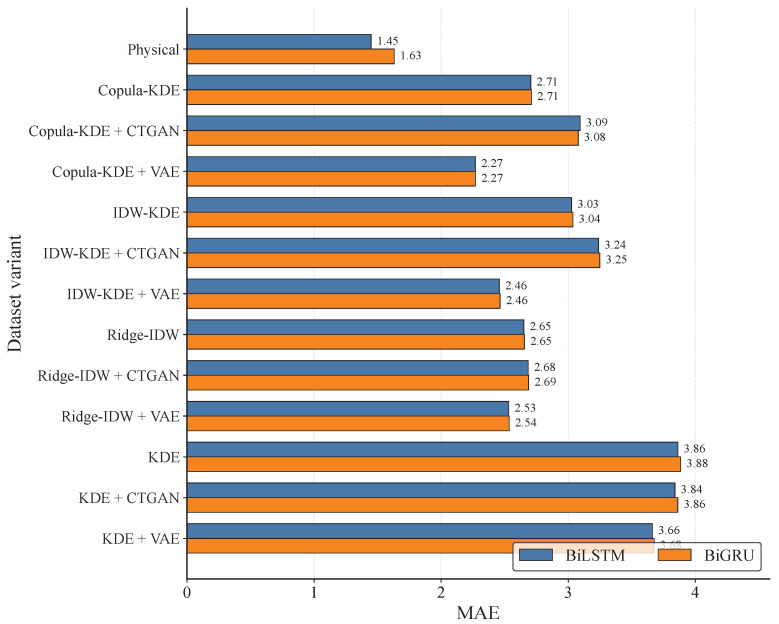
MAE comparison of BiLSTM and BiGRU models for temperature prediction across physical and virtual sensor data variants.

Figure 20 compares temperature prediction performance. The physical sensor data achieves the lowest MAE, with values of 1.45 for BiLSTM and 1.63 for BiGRU, confirming that direct physical measurements remain the most accurate source for temperature modeling. Among the virtual sensor methods, the fused Copula–KDE with VAE achieves the best temperature performance, with MAE values of 2.27 for both BiLSTM and BiGRU. The KL-IDW–KDE with VAE variant also performs well, achieving MAE values of 2.46 for both models. In contrast, the standalone KDE-based methods produce the highest temperature errors, with MAE values above 3.66. These results indicate that fusion combined with VAE augmentation provides a more reliable temperature representation than standalone KDE or CTGAN-based augmentation.

**Figure 21:**
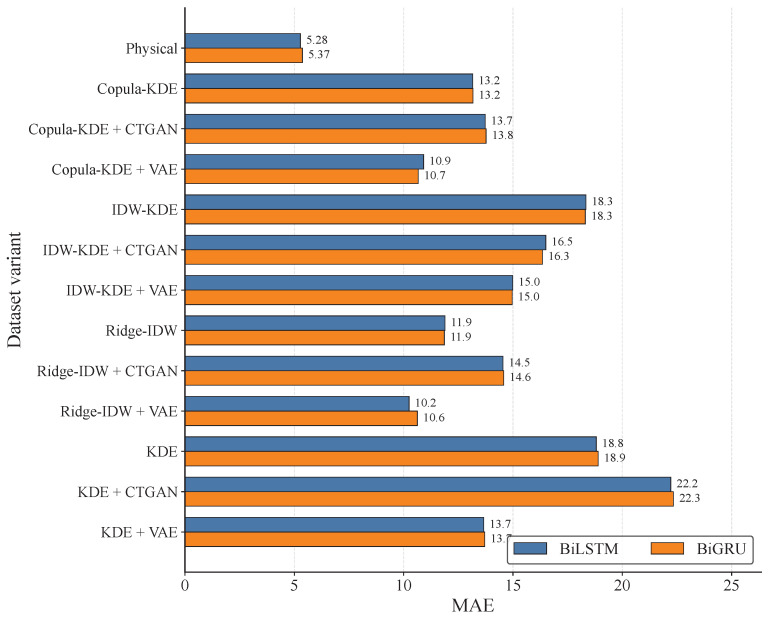
MAE comparison of BiLSTM and BiGRU models for humidity prediction across physical and virtual sensor data variants.

Figure 21 presents the humidity prediction comparison across the physical and virtual sensor data variants. The physical sensor baseline achieves MAE values of 5.28 for BiLSTM and 5.37 for BiGRU, giving the lowest humidity prediction errors among all compared cases. Among the virtual sensor datasets, the KL-Ridge–IDW with VAE variant provides the best BiLSTM result, with an MAE of 10.25, while the fused Copula–KDE with VAE variant achieves the best BiGRU result, with an MAE of 10.67. Overall, VAE-augmented variants show lower humidity errors than their corresponding fused or CTGAN-based versions, indicating that VAE better preserves humidity-related distributional characteristics. Although the virtual sensor variants do not outperform the physical baseline for humidity, the fusion- and VAE-based datasets still provide improved humidity prediction compared with standalone KDE and several CTGAN-based variants.

**Figure 22:**
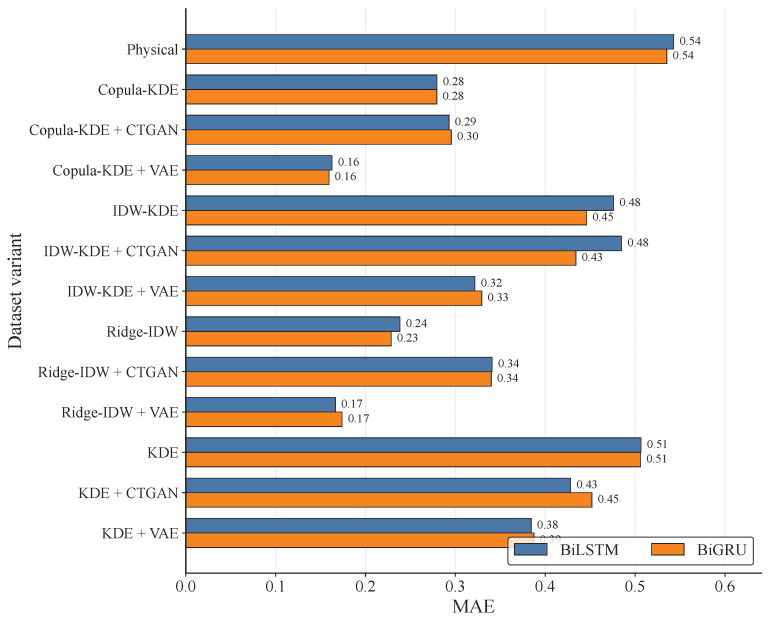
MAE comparison of BiLSTM and BiGRU models for wind direction prediction across physical and virtual sensor data variants.

Figure 22 illustrates the MAE comparison for wind direction prediction. The physical sensor baseline produces MAE values of 0.54 for both BiLSTM and BiGRU. The fused virtual sensor methods substantially reduce this error. The fused Copula–KDE with VAE achieves the lowest wind direction MAE, with values of 0.16 for both models. The KL-Ridge–IDW with VAE variant also performs strongly, with MAE values of 0.17 for both BiLSTM and BiGRU. These improvements are mainly attributed to the sine–cosine circular encoding of wind direction, which reduces the artificial discontinuity at the $0^{\circ }/360^{\circ }$ boundary. Overall, the results demonstrate that the proposed fusion and augmentation strategy significantly improves the modeling of angular sensor variables.

**Figure 23:**
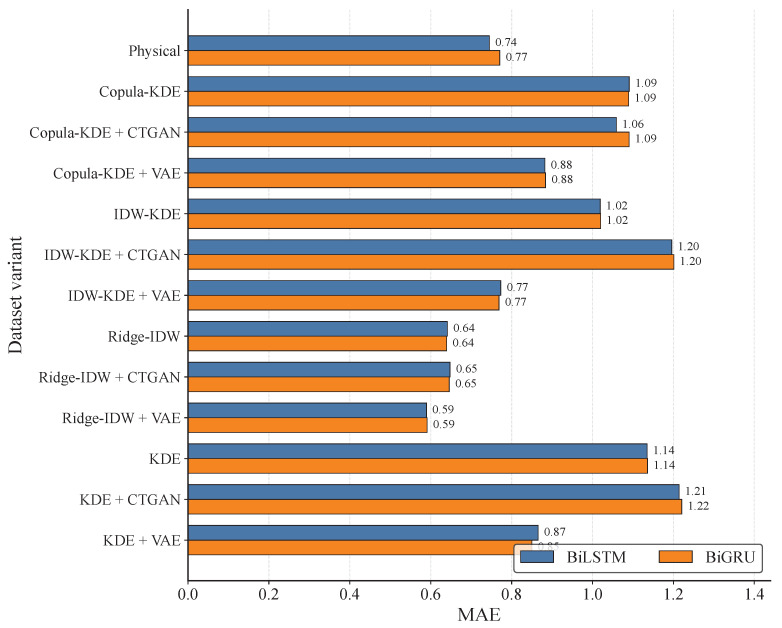
MAE comparison of BiLSTM and BiGRU models for wind speed prediction across physical and virtual sensor data variants.

Figure 23 compares wind speed prediction performance. The physical sensor baseline achieves MAE values of 0.74 and 0.77 for BiLSTM and BiGRU, respectively. Among all virtual sensor methods, the KL-Ridge–IDW with VAE variant achieves the best performance, with MAE values of 0.59 for both models. This is lower than the physical sensor baseline and all other virtual sensor configurations. The KL-Ridge–IDW fused baseline and CTGAN variant also perform competitively, with MAE values around 0.64–0.65. In contrast, standalone KDE and CTGAN-based KDE variants show higher wind speed errors, reaching up to 1.21–1.22. These results confirm that KL-Ridge–IDW fusion, particularly when combined with VAE augmentation, provides the most accurate wind speed representation.

Overall, the numerical results demonstrate that the proposed virtual sensor generation framework improves predictive performance across several environmental variables. Fusion-based methods consistently outperform the standalone KDE approach, confirming the effectiveness of KL-divergence-based fusion. VAE augmentation generally provides more stable and lower prediction errors than CTGAN, especially for humidity, temperature, wind direction, and wind speed. CTGAN can increase sample diversity, but it may also introduce additional distributional variation, which can increase prediction error in some cases. Between the learning models, BiLSTM and BiGRU show comparable performance across most datasets, with BiGRU remaining competitive for wind-related variables and BiLSTM performing strongly for several scalar-variable cases. These findings confirm that the combination of KL-divergence-based fusion, VAE augmentation, and sequence learning provides a reliable framework for high-fidelity virtual sensor generation.

### 4.2. Comparison with Temporal Generative Baselines

To further evaluate the proposed framework against recent temporal generative approaches, additional baseline experiments were conducted using TimeGAN and a diffusion-based time-series generator. In both baselines, synthetic temporal sensor sequences were first generated and then used to train the same downstream BiGRU prediction model. This experimental design provides a fair comparison because all methods follow the same evaluation pipeline: synthetic or virtual sensor data generation followed by BiGRU-based prediction and evaluation using MAE, MSE, and RMSE.

Table 14 compares the proposed framework with temporal generative baselines using BiGRU as the downstream prediction model. Compared with TimeGAN + BiGRU, the proposed KL-Fusion + VAE + BiGRU framework achieves lower MAE values across all evaluated variables. Specifically, the proposed framework reduces the temperature MAE from 4.7075 to 2.2697, humidity MAE from 16.9984 to 10.6668, wind-direction MAE from 1.0545 to 0.1594, and wind-speed MAE from 2.2265 to 0.8839. These improvements indicate that the proposed KL-divergence-based fusion and VAE-based augmentation generate more reliable virtual sensor representations than the TimeGAN-based temporal synthetic data baseline.

Compared with the diffusion-based generative baseline, the proposed framework achieves lower MAE for temperature, wind direction, and wind speed, while the diffusion-based baseline achieves the lowest humidity MAE. However, the proposed framework still substantially reduces humidity error compared with TimeGAN and provides more balanced performance across the evaluated variables. The stronger performance for wind-related variables is particularly important because wind direction and wind speed are more sensitive to circular representation, temporal variability, and distributional shift. Overall, the results show that the proposed KL-Fusion + VAE + BiGRU framework consistently outperforms TimeGAN and achieves competitive performance compared with the diffusion-based baseline, especially for temperature, wind direction, and wind speed. It should also be noted that TimeGAN and diffusion-based temporal generation methods are computationally more expensive than the proposed fusion-based workflow because they require iterative adversarial and denoising-based training before downstream prediction.

### 4.3. Statistical Significance Analysis

To further verify whether the observed performance differences are statistically meaningful, a paired statistical significance analysis was conducted using the Wilcoxon signed-rank test. The test was applied to the absolute prediction errors obtained from the same test samples. This paired testing strategy is suitable because the proposed framework and the baseline models were evaluated on identical test instances. A significance level of p<0.05 was used to determine whether the difference between the paired error distributions was statistically significant.

Table 15 shows the statistical significance results between the proposed framework and the temporal generative baselines. The obtained *p*-values are lower than 0.05 for all evaluated variables, indicating that the differences in prediction error distributions are statistically significant. These results confirm that the performance differences observed in MAE, MSE, and RMSE are not only numerical differences but are also statistically meaningful.

### 4.4. External Validation on a Public NOAA Weather Dataset

To further examine the transferability of the proposed framework, an additional validation experiment was conducted using a public NOAA Local Climatological Data (LCD) weather-station dataset [45]. The dataset was collected from station USW00014826 and contains hourly meteorological observations, including temperature, relative humidity, wind direction, and wind speed. The same preprocessing strategy was applied, including missing-value removal, physical-constraint filtering, unit conversion, wind-direction sine–cosine encoding, KL-Ridge–IDW fusion, and BiGRU-based temporal prediction. This experiment was designed to evaluate whether the proposed virtual sensing workflow remains applicable to an external public dataset beyond the original private sensor data.

Table 16 presents the external validation results on the public NOAA dataset. The KL-Ridge–IDW fused data achieves the lowest temperature MAE of 0.7746, improving over the original public-data baseline MAE of 0.8730. For humidity, wind direction, and wind speed, the fused-data results remain close to the original-data baseline, with MAE values of 3.3330, 11.8946, and 0.3627, respectively. These results indicate that the KL-divergence-based fusion strategy can generate externally valid virtual sensor representations with competitive prediction accuracy on public weather-station data.

The KL-Ridge–IDW + VAE variant shows higher errors on the NOAA dataset, particularly for temperature, humidity, and wind direction. This suggests that VAE augmentation may require dataset-specific tuning when transferred to external datasets with different climatic ranges, sampling characteristics, and station-specific distributions. Overall, the external validation confirms that the proposed fusion-based virtual sensing workflow is transferable to a public dataset, while also highlighting that deep generative augmentation should be carefully calibrated for new sensor domains.

## 5. Conclusions and Future Work

This study proposed an intelligent virtual sensor generation framework for smart environmental monitoring by integrating statistical virtual sensor modeling, physical-constraint validation, KL-divergence-based fusion, deep generative augmentation, and temporal prediction. The framework addresses missing, noisy, and unreliable measurements caused by sparse sensor deployment, sensor faults, calibration drift, and communication interruptions. Physical constraints were applied to ensure that generated values remained within meaningful ranges, including humidity within [0,100], wind speed greater than or equal to 0 m/s, and wind direction within $\left[0^{\circ },360^{\circ }\right)$. In addition, wind direction was encoded using sine and cosine components and reconstructed using atan2, reducing the discontinuity problem at the $0^{\circ }/360^{\circ }$ boundary.

The experimental results demonstrate that KL-divergence-based fusion improves the distributional consistency and predictive usefulness of virtual sensor data compared with standalone generation methods. Among the augmentation methods, VAE generally preserves the original data structure more effectively and provides lower prediction errors, whereas CTGAN offers broader sample diversity but may introduce additional distributional deviations. The BiLSTM and BiGRU evaluations further confirm that the generated virtual sensor data can support reliable temporal prediction, with model performance varying across variables and data-generation configurations. BiGRU remains competitive for wind-related variables, while both BiLSTM and BiGRU provide stable humidity prediction performance depending on the data-generation setting. Additional comparisons with TimeGAN and diffusion-based temporal generative baselines demonstrate that the proposed KL-fusion and VAE-based framework achieves competitive performance, particularly for temperature, wind direction, and wind speed. The Wilcoxon signed-rank test further confirms that the observed performance differences are statistically significant.

To further examine transferability, an external validation experiment was conducted using a public NOAA Local Climatological Data (LCD) weather-station dataset containing temperature, relative humidity, wind direction, and wind speed observations. The results show that the proposed KL-Ridge–IDW fusion workflow remains applicable to public weather-station data and provides competitive prediction accuracy compared with the original public-data baseline. In addition, the computational complexity analysis demonstrates that the proposed framework is practically feasible for medium-scale environmental sensor datasets, with runtime and memory requirements mainly governed by deep generative augmentation and recurrent prediction stages.

Future work will focus on real-time deployment, uncertainty-aware fusion, adaptive reliability estimation, and validation on larger multi-location sensor networks and additional public environmental monitoring datasets. Further studies will also investigate dataset-specific generative model tuning, physics-informed learning, and attention-based temporal models to improve robustness, transferability, interpretability, and practical deployment in smart sensing applications.

## Figures and Tables

**Table 1:** Comparison of recent sensor, temporal prediction, and temporal generative studies with the proposed framework.

Study	Main Focus	Key Strength	Limitation Compared with This Work
Miranda et al. [20]	Virtual sensor modeling	Estimates missing or unreliable measurements	Limited fusion, augmentation, and temporal prediction
Jutras et al. [27]	Copula dependency modeling	Captures multivariate dependencies	No complete augmentation–prediction pipeline
Liu et al. [28]	IDW-based spatial estimation	Effective for spatial sensor estimation	Limited modeling of nonlinear temporal dynamics
Dong et al. [37]	ML-assisted physical sensing	High recognition accuracy	Focuses on physical sensor classification
Ahmed et al. [43]	Synthetic tabular data	Improves data availability	Limited physical constraints and KL-based fusion
Zhou et al. [44]	Transformer forecasting	Captures long-range temporal dependency	Prediction-oriented; no virtual sensor generation
Lim et al. [38]	TFT forecasting	Interpretable multi-horizon prediction	Forecasting-focused, not virtual sensing
Jin et al. [39]	Spatio-temporal GNNs	Models graph-based sensor dependencies	Requires explicit sensor graph topology
Yoon et al. [41]	Temporal data generation	Preserves temporal dynamics	Adversarial training; no physical/KL fusion
Lin et al. [42]	Diffusion-based time-series generation	Strong iterative generative modeling	Computationally expensive denoising process
Proposed framework	Virtual sensor generation and prediction	Unified physical, statistical, generative, and temporal pipeline	Needs tuning and validation for other domains

**Table 2:** Description of physical sensor variables used in the study, including data types and corresponding environmental measurements collected from the weather station.

#	Physical Sensors	Data Type	Description
1	Timestamp	Temporal (datetime)	The date and time when the data was recorded.
2	Temperature	Continuous (float)	Temperature in Celsius (°C) measured at the weather station.
3	Humidity	Continuous (float)	Relative humidity in percentage (%), indicating the amount of moisture in the air.
4	Wind Direction Angle	Continuous (float)	Average wind direction angle in degrees (0–360°). Since this is a circular variable, it is encoded using sine and cosine components for model training.
5	Wind Speed	Continuous (float)	Wind speed in meters per second (m/s), indicating the magnitude of air movement.
6	Longitude	Continuous (float)	Longitude coordinate of the sensor location (in degrees).
7	Latitude	Continuous (float)	Latitude coordinate of the sensor location (in degrees).

**Table 3:** Physical constraints used for validating sensor variables.

Variable	Physical Constraint Used in Validation	Explanation
Temperature	−10≤Temp≤30	According to the region temperature.
Humidity	0≤RH≤100	Relative humidity percentage.
Wind speed	v≥0	Wind speed cannot be negative.
Wind direction	0≤θ<360	Circular angular variable.
Longitude	Valid GPS longitude range/station metadata	Sensor location coordinate.
Latitude	Valid GPS latitude range/station metadata	Sensor location coordinate.

**Table 4:** Experimental system configuration used for model training and evaluation.

Component	Specification
Operating system	Windows 10 for PC Server
Main memory	96 GB RAM
Processor	12th Gen Intel(R) Core(TM) i9-12900K, 3.20 GHz
Programming language	Python 3
IDE	PyCharm Professional 2024.3.5
Data storage/format	CSV and MS Excel files
Core libraries	Pandas 2.3.3, Scikit-learn 1.9.0, Keras 3.14.1, TensorFlow 2.21.0, Seaborn 0.13.2, Matplotlib 3.10.9, CTGAN 0.12.1, and PyTorch 2.12.0

**Table 5:** Key hyperparameters of the VAE and CTGAN models used for data augmentation.

Parameter	VAE	CTGAN
Data split	80% training, 20% validation	80% training, 20% validation
Latent/noise dimension	40	Default CTGAN noise dimension
Network structure	Encoder: Dense(128)–Dense(64); Decoder: Dense(64)–Dense(128)–Dense(input dim.)	Generator dimensions: (128, 128); default discriminator
Activation function	ReLU in hidden layers	Default CTGAN activations
Loss function	Reconstruction MSE + KL-divergence	Adversarial training loss
KL weighting	Linear warm-up: min(1.0,epoch/50)	Not applicable
Optimizer	Adam	Adam-based CTGAN optimizer
Learning rate	0.001	Default CTGAN learning rate
Batch size	16	32
Epochs	50	50
Output validation	Inverse scaling and physical validity filtering	Inverse scaling and physical validity filtering

**Table 6:** Key network structure and training parameters of the BiLSTM and BiGRU models.

Parameter	BiLSTM	BiGRU
Input sequence length	3 time steps	3 time steps
Input shape	(3,1) for scalar features; (3,2) for WindDirX/WindDirY	(3,1) for scalar features; (3,2) for WindDirX/WindDirY
Recurrent layers	BiLSTM(64, ReLU, return sequences) → BiLSTM(32, ReLU)	BiGRU(64, ReLU, return sequences) → BiGRU(32, ReLU)
Dropout	0.3 after first recurrent layer; 0.2 after second recurrent layer	0.3 after first recurrent layer; 0.2 after second recurrent layer
Dense layer	Dense(32, ReLU)	Dense(32, ReLU)
Output layer	Dense(1) for scalar features; Dense(2) for WindDirX/WindDirY	Dense(1) for scalar features; Dense(2) for WindDirX/WindDirY
Optimizer	Adam	Adam
Learning rate	0.001	0.001
Loss function	MSE	MSE
Epochs	50	50
Batch size	32	32
Train-test split	80% training, 20% testing	80% training, 20% testing
Wind direction reconstruction	atan2(WindDirY, WindDirX), mapped to $\left[0^{\circ },360^{\circ }\right)$	atan2(WindDirY, WindDirX), mapped to $\left[0^{\circ },360^{\circ }\right)$

**Table 7:** Computational characteristics and scalability considerations of the proposed framework.

Stage	Main Operations	Computational Cost	Scalability Consideration
Preprocessing	Missing-value handling, physical constraints, scaling, wind-direction encoding	Low; approximately linear with data size	Easily scalable using batch or streaming preprocessing
Virtual sensor generation	IDW, KDE, Ridge-based estimation, Copula-based modeling	Moderate; depends on records, variables, and estimation method	Can be parallelized across variables or sensor locations
KL-divergence fusion	Distributional comparison and best-source selection	Moderate; depends on number of candidate virtual sensors and features	Scalable through feature-wise or location-wise computation
VAE/CTGAN augmentation	Iterative deep generative model training and sample validation	High; requires repeated neural network optimization	Can use mini-batch training, GPU acceleration, and parallel runs
BiLSTM/BiGRU prediction	Sequence construction and recurrent neural network training	Moderate to high; depends on sequence length, epochs, and batch size	Scalable using shorter windows, mini-batches, and model compression
Evaluation	MAE, MSE, RMSE, and wind-direction reconstruction	Low	Easily scalable and can be automated

**Table 8:** Quantitative computational cost and scalability analysis of the main augmentation and temporal prediction components.

Component	Dataset/Case	Training Time (s)	Inference Time (s)	Single Inference Time (s)	Memory Increase (MB)	Time Complexity
CTGAN augmentation	CTGAN data	351.79	0.054	–	150.43	O(Endh)
VAE augmentation	VAE data	1077.27	0.003	–	12.52	O(Endh)
BiLSTM prediction	Original data	1928.40	61.11	0.1154	150.21	$O(EnTh^{2})$/$O(n_{\mathrm{test}}Th^{2})$
BiGRU prediction	Original data	1343.79	37.58	0.0709	72.28	$O(EnTh^{2})$/$O(n_{\mathrm{test}}Th^{2})$
BiLSTM prediction	KL-Ridge-IDW data	115.72	72.48	0.1481	64.02	$O(EnTh^{2})$/$O(n_{\mathrm{test}}Th^{2})$
BiGRU prediction	KL-Ridge-IDW data	66.33	34.53	0.0719	73.96	$O(EnTh^{2})$/$O(n_{\mathrm{test}}Th^{2})$
BiLSTM prediction	Copula-KDE + VAE data	42.45	30.32	0.0643	59.27	$O(EnTh^{2})$/$O(n_{\mathrm{test}}Th^{2})$
BiGRU prediction	Copula-KDE + VAE data	74.68	55.15	0.1125	68.00	$O(EnTh^{2})$/$O(n_{\mathrm{test}}Th^{2})$

**Table 9:** Experimental Results of BiLSTM and BiGRU Models on Physical Sensors Data.

Feature	BiLSTM			BiGRU		
	MAE	MSE	RMSE	MAE	MSE	RMSE
temperature	1.448813	4.143780	2.035628	1.630625	4.904090	2.214518
humidity	5.279900	70.627216	8.404000	5.370100	73.673039	8.583300
winddirangleavg	0.543000	0.431800	0.657100	0.535280	0.430390	0.656040
windspeedmax	0.744917	0.923897	0.961196	0.770717	0.957817	0.978682

**Table 10:** Experimental Results of BiLSTM and BiGRU Models on Fused Copula-KDE Weighted Virtual Sensor Data Variants.

Sensors	BiLSTM			BiGRU		
	MAE	MSE	RMSE	MAE	MSE	RMSE
Experimental Results of a Learning Model with Fused Copula KDE Data						
temperature	2.705277	11.718570	3.423240	2.710848	11.843070	3.441376
humidity	13.161027	249.928821	15.809137	13.170420	249.856166	15.806839
winddirangleavg	0.279480	0.118100	0.343780	0.279350	0.119400	0.345650
windspeedmax	1.090956	1.691932	1.300743	1.089263	1.698534	1.303278
Experimental Results of a Learning Model on Fused-CTGAN Data						
temperature	3.094070	14.901010	3.860182	3.078674	14.814790	3.848999
humidity	13.732957	272.891302	16.519422	13.773016	275.639974	16.602409
winddirangleavg	0.292930	0.146370	0.382580	0.295500	0.144030	0.379500
windspeedmax	1.059140	1.746934	1.321716	1.090574	1.792604	1.338881
Experimental Results of a Learning Model on Fused-VAE Data						
temperature	2.269028	7.944535	2.818605	2.269672	7.936787	2.817230
humidity	10.913837	183.663820	13.552263	10.666777	177.631780	13.327857
winddirangleavg	0.162810	0.046050	0.214590	0.159390	0.045280	0.212800
windspeedmax	0.882243	1.048907	1.024162	0.883929	1.051384	1.025370

**Table 11:** Experimental Results of a Learning Model for Virtual Sensors based on KL-IDW-KDE, CTGAN, and VAE Data.

Sensors	BiLSTM			BiGRU		
	MAE	MSE	RMSE	MAE	MSE	RMSE
Experimental Results on KL-IDW-KDE Fused Virtual Sensor Data						
temperature	3.026100	13.894600	3.727500	3.036400	14.062800	3.750000
humidity	18.334388	462.720927	21.510949	18.310038	462.794497	21.512659
winddirangleavg	0.475960	0.361058	0.600800	0.445960	0.370340	0.608500
windspeedmax	1.019100	1.492500	1.221700	1.020200	1.489300	1.220400
Experimental Results on KL-IDW-KDE with CTGAN Data						
temperature	3.238600	16.077100	4.009600	3.247900	16.128900	4.016100
humidity	16.502866	425.539386	20.628606	16.349276	414.968922	20.370786
winddirangleavg	0.484890	0.364200	0.603490	0.434080	0.384550	0.620100
windspeedmax	1.195900	1.818700	1.348600	1.201300	1.828800	1.352300
Experimental Results on KL-IDW-KDE with VAE Data						
temperature	2.458100	9.835900	3.136200	2.463700	9.804000	3.131100
humidity	14.986074	334.733370	18.295720	14.966607	334.624520	18.292745
winddirangleavg	0.321710	0.189690	0.435500	0.329310	0.184870	0.429960
windspeedmax	0.773200	0.898700	0.948000	0.768800	0.909900	0.953900

**Table 12:** Experimental Results of a Learning Model for Virtual Sensors based on KL-Ridge-IDW, CTGAN, and VAE Data.

Sensors	BiLSTM			BiGRU		
	MAE	MSE	RMSE	MAE	MSE	RMSE
Experimental Results on KL-Ridge-IDW Fused Virtual Sensor Data						
temperature	2.650400	10.764100	3.280900	2.654300	10.777700	3.282900
humidity	11.891702	223.552805	14.951682	11.864079	223.465279	14.948755
winddirangleavg	0.238300	0.100500	0.317040	0.228580	0.104800	0.323770
windspeedmax	0.641200	0.656500	0.810300	0.639300	0.665800	0.816000
Experimental Results on KL-Ridge-IDW with CTGAN Data						
temperature	2.684300	11.258600	3.355400	2.688100	11.377300	3.373000
humidity	14.544929	309.811054	17.601450	14.574742	312.976560	17.691144
winddirangleavg	0.340970	0.181320	0.425810	0.339930	0.179650	0.423860
windspeedmax	0.647700	0.635700	0.797300	0.645800	0.630200	0.793900
Experimental Results on KL-Ridge-IDW with VAE Data						
temperature	2.531300	10.184400	3.191300	2.535000	10.237600	3.199600
humidity	10.248988	166.336333	12.897144	10.628155	176.619255	13.289818
winddirangleavg	0.166470	0.048700	0.220750	0.173850	0.050348	0.224380
windspeedmax	0.589600	0.495200	0.703700	0.591100	0.496000	0.704300

**Table 13:** Experimental Results of a Learning Model for Virtual Sensors based on KDE, KDE-CTGAN, and KDE-VAE Data.

Sensors	BiLSTM			BiGRU		
	MAE	MSE	RMSE	MAE	MSE	RMSE
Experimental Results on KDE Virtual Sensor Data						
temperature	3.863000	23.701600	4.868400	3.883900	24.005900	4.899600
humidity	18.812860	490.288933	22.142469	18.898043	497.731995	22.309908
winddirangleavg	0.506560	0.417130	0.645860	0.505970	0.416330	0.645236
windspeedmax	1.135000	1.831800	1.353400	1.136000	1.828100	1.352100
Experimental Results on KDE with CTGAN Data						
temperature	3.839900	23.665100	4.864700	3.861900	23.962200	4.895100
humidity	22.217714	699.933905	26.456264	22.335684	704.135255	26.535547
winddirangleavg	0.428180	0.299130	0.546930	0.451960	0.307280	0.554330
windspeedmax	1.214100	2.062900	1.436300	1.220600	2.112100	1.453300
Experimental Results on KDE with VAE Data						
temperature	3.662600	19.831200	4.453200	3.676400	20.068100	4.479700
humidity	13.663877	270.809119	16.456279	13.707969	272.862230	16.518542
winddirangleavg	0.384350	0.228350	0.477860	0.387490	0.230820	0.480440
windspeedmax	0.865700	1.010500	1.005200	0.850200	0.996100	0.998100

**Table 14:** Comparison with temporal generative baselines using BiGRU as the downstream prediction model.

Method	Temperature MAE	Humidity MAE	Wind Direction MAE	Wind Speed MAE
TimeGAN	4.7075	16.9984	1.0545	2.2265
Diffusion	2.4285	3.9679	0.6157	0.8915
Proposed	2.2697	10.6668	0.1594	0.8839

**Table 15:** Statistical significance analysis using the Wilcoxon signed-rank test.

Comparison	Variable	*p*-Value	Significance
Proposed vs. TimeGAN	Temperature	$4.05\times 10^{-34}$	Significant
Proposed vs. TimeGAN	Humidity	$2.05\times 10^{-20}$	Significant
Proposed vs. TimeGAN	Wind direction	$4.82\times 10^{-28}$	Significant
Proposed vs. TimeGAN	Wind speed	$1.48\times 10^{-3}$	Significant
Proposed vs. Diffusion	Temperature	$1.08\times 10^{-15}$	Significant
Proposed vs. Diffusion	Humidity	$8.74\times 10^{-32}$	Significant
Proposed vs. Diffusion	Wind direction	$5.29\times 10^{-28}$	Significant
Proposed vs. Diffusion	Wind speed	$3.53\times 10^{-3}$	Significant

**Table 16:** External validation using the public NOAA LCD weather-station dataset with BiGRU as the downstream prediction model.

Dataset/Method	Temperature MAE	Humidity MAE	Wind Direction MAE	Wind Speed MAE
Original NOAA Public Data	0.8730	3.2324	11.7888	0.3593
KL-Ridge–IDW Fused NOAA Data	0.7746	3.3330	11.8946	0.3627
KL-Ridge–IDW + VAE NOAA Data	5.2698	11.6837	61.5136	0.7058

## Data Availability

Due to project-level data-use and sharing restrictions, the raw private dataset, full source code, and trained models cannot be publicly released at this stage. However, detailed implementation settings, preprocessing steps, model configurations, hyperparameters, and evaluation procedures are provided to support reproducibility. Processed data, scripts, and trained models will be available from the corresponding author upon reasonable request, subject to project approval. The public NOAA Local Climatological Data (LCD), Version 2 dataset used for external validation is available from the NOAA National Centers for Environmental Information at https://www.ncei.noaa.gov/products/land-based-station/local-climatological-data (accessed on 24 June 2026).
